# Stress, Burnout, and Turnover Issues of Black Expatriate Education Professionals in South Korea: Social Biases, Discrimination, and Workplace Bullying

**DOI:** 10.3390/ijerph17113851

**Published:** 2020-05-29

**Authors:** Luis Miguel Dos Santos

**Affiliations:** Woosong Language Institute, Woosong University, Daejeon 34514, Korea; luisdossantos@woosong.org; Tel.: +82-010-3066-7818

**Keywords:** burnout, career decision, discrimination, foreign professionals, social biases, South Korea, stress, suicide, turnover, workplace bullying

## Abstract

The purpose of this study is to understand expatriate educators’ overall teaching experiences and opinions about living in South Korea. The research study sought to explore the relationship between stressors and social biases against Black individuals and their suicidal behaviours and turnover decisions. The approach of stress, burnout, career decision, and suicide guided this study as the lens. The research method for this study included a phenomenological analysis of two sessions of semi-structured interviews with 18 Black expatriates in the field of education in South Korea. The results indicate that their experiences were impacted by unfairness against individuals based on their skin colour and nationalities. The outcomes of this study highlight the major difficulties experienced by foreign professionals living in South Korea. They can be used by human resource professionals, school administrators, and government leaders to reform their current policy and improve expatriate experiences so as to prevent turnover.

## 1. Introduction

Stress is not an isolated situation in a particular region but an international problem at local schools, international schools, universities, private teaching academies, and even community centres [1]. Education is one of the fields in which people face high levels of stress. Such stressful working environments cause frequent departures, burnout, and mental distress [2]. As a result, stress is usually considered to be a major cause of education professionals leaving their jobs and the resultant shortage of staff, not only because of the problem in hiring appropriate qualified professionals but also because of the issue of retaining them [3]. As an international perspective, a previous study [4] has indicated that more than 30% of freshly graduated licensed teachers and education professionals from universities decide to leave the teaching profession after their placement internships [5]; another 30% decide to leave after a few years into their contracts [6]. In other words, as an international practice, a large number of teachers decide to leave the teaching profession after a few years of the teaching career path [5].

Although many school leaders and policymakers have established alternative plans, such as increasing the number of school professional staff and clerks for administrative work, stress is still a concern for teachers and education professionals [7]. Many teachers and education professionals describe themselves as being confused, concerned, overloaded, sad, and angry. Due to the additional personal, mental, and emotional concerns brought about by their working environment [8], some teachers and education professionals have blamed themselves for not being considerate towards their students. A previous study indicated that some teachers and education professionals might experience burnout, stress, and mental distress (e.g., suicidal ideation or suicidal behaviour) due to various factors and elements [9].

Significantly, some teachers and education professionals may associate with mental problems due to high levels of stress, burnout, workplace bullying, social and cultural biases, and discrimination from both internal (i.e., school) and external (i.e., society) environments. Research [10] has indicated that social expectations, stress, discrimination, and burnout are some reasons for people committing suicide or related issues. Therefore, education professionals in international schools and universities who come from non-South Korean social and cultural backgrounds could have difficulties in handling stress originating from society.

### 1.1. General Research Background

During the past decade, many school districts, governmental agencies, and universities have advocated that teaching and education should receive special focus, in accord with the communities’ long-term investments and efforts towards sustainable development [11]. As a result, governmental agencies, NGOs, and teachers’ professional training centres have developed a series of traditional routes, alternative approaches, and assessment-only teacher-training programmes to address the shortage of qualified teachers, nurses, and counsellors (i.e., education professionals) for the next generation. However, although such teacher-training programmes have been established and applied, human resource problems have not been solved. 

In East Asia, rapid development has prompted the South Korean government to recruit foreign professionals, researchers, and scholars from foreign countries to come to the region for social, cultural, and scientific research and development because the region relies heavily on experts and scholars with top-tier and international experiences [12]. Therefore, since the South Korean educational system often is not suitable for international qualifications (e.g., the International Baccalaureate), there is a particular need to provide the region with international schools, universities, and educational programmes [5].

As of late 2019, there are 40 international schools in South Korea, offering instruction in English, Japanese, German, Mongolian, French, and Chinese from the preschool to secondary school levels, and more than 220 public and private universities throughout the region. As the international schools and universities exist to provide international and university-level curricula and multiple language backgrounds, most of them have had to recruit qualified education professionals from their home countries, with the particular need to acquire experienced teachers and education professionals with industry and professional practices.

However, international schools and universities face difficulties with recruitment and frequent turnover, due to a low level of job satisfaction, burnout, workplace bullying, negative administrative environments, psychological stress, and even discrimination due to both internal elements (i.e., issues inside the school) and the external elements (i.e., issues from social and cultural environments) of the host countries and regions [13]. Although different school districts and geographic regions face different issues, most of them encounter the same problems, especially common difficulties with recruitment and frequent turnover. School leaders have consistently supported educational staff’s professional development and human resources planning in an effort to engage the education professionals’ sense of belonging and satisfaction in their teaching environments. However, many education professionals continue to leave South Korea for other countries [14]. 

### 1.2. Purpose of the Study: The Focus of Black Education Professionals

This study has three purposes. First, it seeks to understand and explore foreign secondary school and university education professionals’ overall teaching experiences and their opinions about living in South Korean society, particularly Black education professionals. South Korea is a popular destination for teaching in international schools and universities because of the scarcity of qualified education professionals in the region. Therefore, international schools and universities routinely host recruitment fairs and virtual recruitment sections to attract qualified teachers and education professionals to their human resource pools. However, most of the international schools and universities continued to face the frequent departure of their qualified personnel. From the perspectives and lens of the approach of stress, burnout, career decision, and suicide, the researcher would like to seek and explore the understanding, life stories, and situations of a group of in-service and post-service international schools and universities education professionals in South Korea, particularly Black education professionals.

The second purpose of this study is to explore the living and teaching experiences of Black education professionals in the South Korean environment. The results focus on two directions (i.e., internal and external elements) based on the direction of the approach of stress, burnout, career decision, and suicide. There are no recent research studies with a focus on Black teachers and education professionals’ living and teaching experiences in South Korea. Therefore, the outcomes of this study provide a new lens for these issues.

**Figure 1:**
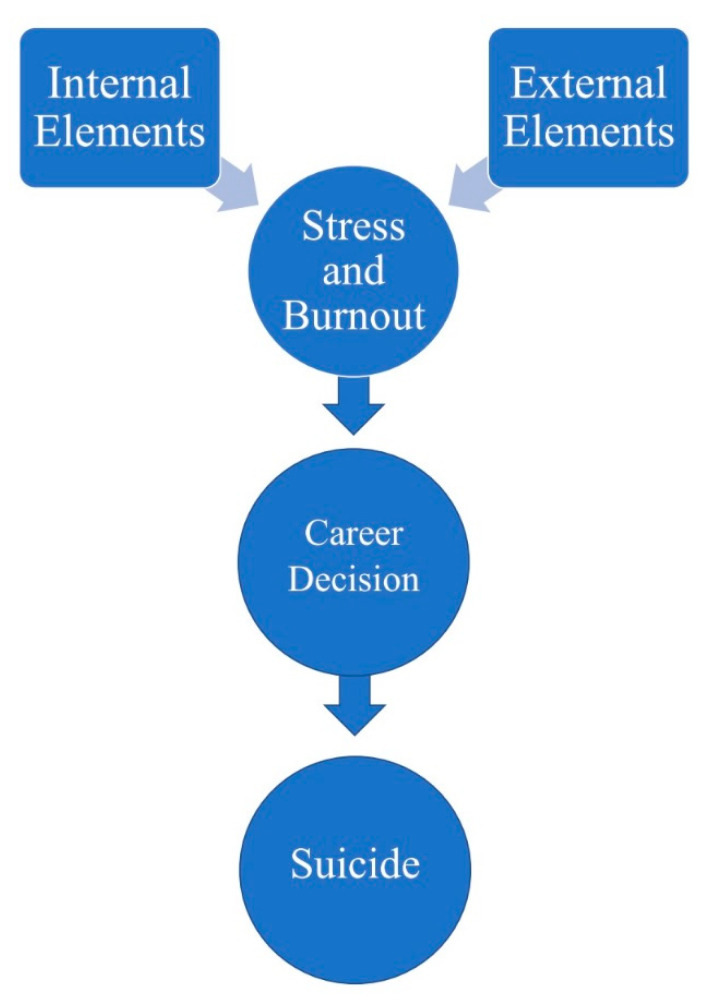
The approach of stress, burnout, career decision, and suicide.

The third purpose of this study is to explore the relationships between the stresses and social and cultural biases against Black international secondary school and university education professionals in South Korea and those individuals’ suicidal behaviour and turnover decisions. More particularly, the researcher tends to seek the understanding and holistic picture of what internal elements at the school level (e.g., administrative styles), and external elements at the social level (e.g., social and cultural biases and discriminations) have influenced their experiences in both South Korean international schools and universities and the South Korean living environment. Figure 1 refers to the perspectives and lens for this study. 

### 1.3. Brief Explanation of the Approach of Stress, Burnout, Career Decision, and Suicide

The approach of stress, burnout, career decision, and suicide was developed by the researcher based on this research study. This approach has three stages. As to the first stage, the approach tries to understand how internal and external elements influence the stress and burnout experiences of individuals and explore what these elements are. As to the second stage, the approach explores the relationship between individuals’ career decisions and the sources of stress and burnout. In other words, how would these sources of stress and burnout behaviours influence the individuals’ career decisions and development? As to the third stage, some individuals may further develop mental distress (e.g., suicidal behaviour) due to the sources of stress and burnout and career decisions and development based on the internal and external elements. 

## 2. Materials and Methods

### 2.1. Background of the Participants

This study reports on some meaningful findings regarding the issues of workplace stress, mental health, and career outcomes of Black education professionals in South Korean international school and university environments based on the approach of stress, burnout, career decision, and suicide, and the lens of social biases, discrimination, and workplace bullying. With the phenomenological analysis [15], the researcher collected data information from 18 Black school professionals. They are currently working or have left (i.e., in-service and post-service) the international school and university environments in South Korea.

All 18 participants were willing to join this research study. More importantly, this study intended to seek out the stress, burnout, career decision, and suicide issues of education professionals in the international school environment in South Korea. Briefly, 61% of the participants were teachers, 22% of the participants were nurses, and 17% of the participants were counsellors. Of the participants, 66% were female individuals and 44% of the participants were male individuals. As to nationalities, 44% of the participants came from North America, 28% of the participants came from Africa, 17% of the participants came from Oceania, and 11% of the participants came from Europe. Table 1 outlines the demography.

The participants were required to meet the following criteria, which were

1. Either in-service or post-service professionals at one of the international school and university environments in South Korea;
2. Providing or used to provide services in the field of education, health, and social caring (i.e., teachers, nurses, and counsellors) in South Korea;
3. Experienced stress, burnout, or mental distress from their experiences in South Korea;
4. Have or had the suicidal behaviour due to the situations in South Korea;
5. Willing to share the truth, real life stories, and experiences for academic purposes;
6. Be a Black person, regardless of gender;
7. Be a non-Korean (i.e., foreigner).

### 2.2. Data Collection

Based on the research purposes and directions, the researcher developed an interview proposal in order to explore the answers [16,17,18]. The researcher invited 18 Black participants to two sessions of open-ended, semi-structured, and individual interviews [19,20]. Please see Appendix A for the interview protocol. All participants were asked the same interview protocols and interview questions. Each session lasted from 62 to 81 min.

The snowball sampling strategy was employed for the invitation. Based on personal networks, the researcher invited five participants for the research study. Each participant was asked to invite additional participants within the targeted criteria. After several rounds of invitations, the researcher invited 18 participants for the research study.

The researcher contacted each potential participant by email invitation [17,21]. The invitation letter provided the information, including the nature, objective, aim, and methodology, requirement of participants, and purpose of the study, with a declaration about their voluntary participation or nonparticipation.

### 2.3. Data Analysis

Themes, directions, and categories that merged during the interview sessions were individually listed. The general inductive approach [22] was employed for data analysis. The general inductive approach allowed the researcher to understand the study of the data information from the interview transcripts. First, the researcher followed the general inductive approach to narrow down the massive-sized interview transcripts and to share the first-level themes and groups by employing an open-coding technique based on the direction of the grounded theory approach. In fact, as the interview protocols and interview questions tended to capture rich and in-depth information from the participants, more than 500 pages of transcripts were merged. After the open-coding technique [16,18], the researcher categorised 25 themes and 21 subthemes for first-level reporting. 

Second, based on the first-level themes and subthemes, the researcher believed that further narrowing had to be conducted and categorised. Therefore, the researcher continued to follow the axial-coding technique for second-level categories [16,18,23]. As a result, two themes and four subthemes were categorised.

### 2.4. Human Subject Protection and Ethical Consideration 

Due to limited networking in South Korea and other East Asian regions, the participants’ confidentiality is of the greatest concern. The researcher has made every effort to protect the participants’ personal information. Therefore, the researcher arranged for each participant to have a pseudonym. Due to the content form and agreement, the researcher needed to mask the names and locations of the schools, nationalities, ages, and years of experience of the participants. However, based on the content form and agreement, the participants were willing to share their position, gender, and place of origin (continent). Some may argue that the information of the participants is important. However, the researcher advocates that the shared experiences of the participants cannot be influenced based on their personal backgrounds. Therefore, the researcher analysed the transcript information as a whole.

All signed and unsigned content forms and agreements, personal contacts, audio recordings, transcripts, computers, and related materials were locked in a password-protected cabinet. Only the researcher has the right to read the information. After the data analysis progression, the researcher destroyed and deleted the materials due to the reasons of privacy. All subjects gave their informed consent for inclusion before they participated in the study. The study was conducted in accordance with the Declaration of Helsinki, and the protocol was approved by the Ethics Committee of The Social and Health Care Centre of South Korea (Summer/Fall/2019).

## 3. Results and Discussions

Surprisingly, all 18 participants expressed negative concerns about the external environment in South Korea’s communities due to social biases and discrimination. Although most of the participants affirmed that the internal elements (i.e., school management and managerial style) were mixed and they had supportive assistance from the school administrators, all 18 had decided to leave the region or school after the current academic term or had already left. Through transcripts, the participants shared their negative experiences: 178 instances of *general discrimination*, 132 instances of *skin colour bias*, 119 occasions of *impolite and rude behaviour*, and 56 times when they had *feelings of hating South Korea*. 

It is worth noting that one of the recruitment criteria of this study was having or have had suicidal behaviour due to the situations in South Korea. However, based on their contribution, only a few participants indicated their suicidal behaviour and life stories. Although the researcher asked the follow-up questions about suicidal behaviour or life stories, only a few participants indicated that they were affected. As a result, although all participants were associated with previous suicidal behaviour, the intentions and motivations were not significant based on the approach of stress, burnout, career decision, and suicide. In other words, most of the participants’ statuses remained at the second stage of the approach of stress, burnout, career decision, and suicide.

Based on the approach of stress, burnout, career decision, and suicide and the data analysis process, in this qualitative study, the researcher completed an inductive analysis of the data and categorised the results into structured themes and subthemes. Table 2 refers to the themes and subthemes of the study.

### 3.1. Internal Stress: Unfairness towards Foreign Professionals 

Expectations from parents, students, university admission officers, potential employers, and even governmental agencies are high in the South Korean educational system [1,24,25]. Due to the perspectives of Chinese influences (i.e., Confucianism) and social expectations [26,27,28], many South Korean people want their children and next-generations to go to a top-tier university and work as a governmental worker after graduation, particularly for families that spend additional tuition fees for international schools and universities. It is not uncommon for international school and university education professionals to face a high level of stress and burnout from these types of school environments [29]. In this study, most participants expressed that their stress and burnout were from the parents, co-workers and administrators, particularly from personnel with South Korean backgrounds, and one said,

*…it is understandable to have high expectations from their children as they are currently enrolled at one of the best international schools in South Korea…but teachers are still a human being, we deserved respectfulness…we are not animal and machine…yes, I received the salary from the parents and school…but I have human rights…I am a teacher, and I am a human…I experienced many discriminations from South Korean people…*(Participant #14, Counsellor, Female)

#### 3.1.1. Stress and Burnout Based on Parental Behaviour

Although schools and educational environments are places that always encourage fairness and social justice, workplace bullying and unfairness from those working environments are not uncommon [30,31]. A previous study [32] has indicated that international school professionals experienced discrimination and social bias due to their nationalities, skin colour, languages, and even religious practices [6]. However, the unfairness and discrimination from parents’ perspectives were vital and significant based on the narration of what happened [33]. For example, all participants experienced verbal harassment from the parents due to their skin colour, and one said,

*…I am a club leader of the Bible Club…an afterschool programme…we have 25 students and we always invited parents to our club bi-monthly…but at least 15 parents called the school administrators…asked their children to leave my club…I asked why…it is because of my skin colour and my nationality as a South African…it was so stressing and I want to die…* (Participant #13, Teacher, Female)

The sharing from this participant echoed [34], where the racial and ethical factors have always influenced the outcomes and experiences of non-South Korean or non-East Asian expatriates and international professionals in South Korea. The discrimination from parents was not a single case, all other education professionals expressed their situation during the open-ended interviews; for example, one said that due to her skin colour, some parents called for the school principal to fire her. The negative working environment and workplace bullying caused a high level of stress and burnout, and she said,

*…it was a very stressful situation…several parents called my school principal to fire me because I am not a White teacher…they want their children to be taught by White teachers with beautiful hair colour…I cried almost every day as I don’t know when will I lose my job…it was a very ugly time of my life...* (Participant #10, Teacher, Female)

Besides the relationship between social biases and professionalism based on their skin colour, many South Korean parents asked the participants to clean up the toilets and restrooms due to their skin colour. For example, eight participants expressed that during the Parents’ Open Day, some parents asked them to clean up the restrooms and toilets as they are Black people. One participant said,

*…it was a workplace bullying and discrimination for sure…many parents and students asked me to clean up the restroom as I am a Black teacher…Black people do not equal to dirty…some Black people work for some professions…but it does not mean all Black people work in that place…I cried all the time after I took this job in South Korea…I want to leave and I hate this working environment…* (Participant #9, Teacher, Female)

#### 3.1.2. Stress and Burnout Based on Behaviour from Co-Workers and Administrators

In the workplace, most of the participants expressed that although the financial and physical contributions (i.e., salary payments, office environments, and classroom equipment) were fair to all people, all participants believed that workload and psychological unfairness to foreign education professionals were the key problems in workplace bullying [35], particularly for Black school professionals [6]. First, many participants expressed that their Korean administrators and leaders always asked them to do additional work and courses. Even though there was no directed and written evidence to show how such unfairness was contributed, many felt the unfairness and discrimination, and one said,

*…I cannot say my Korean supervisor treated me extremely bad or negative…but I can tell that White people, Korean people, and other East Asian co-workers…they have better environments or situations. But for African, Indian, Filipinos and so on…not just me, but we can tell the differences* (Participant #7, Counsellor, Male)

The narration echoed a recent research study [36] about ethical and gender discrimination at international schools in South Korea. The research indicated that minorities and females always face different levels of discrimination and workplace bullying due to some unchangeable factors (e.g., gender and skin colour). In addition to internalised feelings of distress, nearly two-thirds experienced verbal conflict from their Korean school administrators and leaders [37]. The researcher categorised a significant remark from a participant about the verbal harassment from a Korean leader.

*…my school supervisor and administrator called me a Black Monkey in front of a group of parents during the open day…in the Korean language…I understood the Korean word, but what can I do? I asked them to stop this…but the parents all looked at me and laugh at me…am I look monkey…I needed to see a counsellor afterwards…* (Participant #12, Counsellor, Male)

In short, school leaders, co-workers, and parents’ expectations, discrimination, social biases, and racial discrimination served as some sources of stress and burnout of these Black education professionals in South Korea [35]. Such significant sources of stress and burnout became the reasons why Black education professionals would decide to leave their career path [6]. 

### 3.2. External Stress: Discrimination Against the Participants from Members of the General Public 

South Korean society has always promoted itself as a welcoming community with an international perspective and living environment [38,39,40]. However, none of the participants agreed that categorisation, based on their experiences in the region. In the interviews, almost all of the participants’ opinions and life stories were negative. On the topic of social stress and pressure, many pointed out that South Korea has one of the highest suicide rates of OECD countries, due to unbalanced social expectations of both local residents and international professionals [41]. 

#### 3.2.1. Taking Away Employment Opportunities and Disapproval Towards Foreigners

Work-related pressure and expectations are high for many individuals in the South Korean environment. However, because of its lack of experienced professionals in most fields, South Korea is forced to recruit a large number of individuals to work in various companies and organisations, including international schools [42]. Although privacy protection prevents the release of official statistics from human resources management, it is easy to believe that more than half of the country’s international school education professionals are foreign professionals with various backgrounds, skin colour, and nationalities [43].

The participants in this study were all Black education professionals from North America, Europe, Oceania and Africa, so their ethnicity was different from that of almost all of the local Korean people. Among other examples, all of them needed to attend conferences and professional training hosted by some NGOs and government agencies. During those meetings, many were asked by local Korean professionals about their employment opportunities in South Korea. Several shared that the local professionals habitually inquired about them departing their teaching position because of their nationality and background. As one described,

*…Some teachers asked me when will I leave Korea?...They believed we are not Korean and should not work in Korea because the teaching jobs should belong to Korean people…not foreigners…we should not take away Koreans’ jobs, because we are foreigners…such conversations were very impolite and rude, as my employment is not a part of [their] business…* (Participant #4, Teacher, Female)

Many others reported the same ideas. Unlike other groups of people with ideas of individualism [44,45] and personal privacy, Korean people like to seek information on the private backgrounds and personal lives of others for comparison and gossip. Therefore, participants from westernised countries, such as the United States and Canada, disliked any conversations about their salaries and personal life stories. For example, one participant, who experienced discrimination because she did not share her salary with her Korean peers, said,

*…salary is a private matter…I have my rights to hide my personal privacy. But Korean people are so rude…not just one, but all…they always ask my salary, age, marriage status and so on during the dinner and meal time…I shared my marriage status…I am divorced. Next week, almost everyone in the group knew I am a mother with a kid…this is not a single case, gossip is a very common practice in Korea…* (Participant #18, Nurse, Female)

Others shared that the local Korean people always asked them about their salary and the duration of their allotted vacation for comparison with their own situations. Although none of the participants were willing to share that information, the Koreans forced them to and were verbally offensive. A previous study [46] indicated that Korean people like to judge people based on their earnings and positions. Although foreigners may not understand such cultural conflicts and conversations, South Koreans have always forced their thinking and expectations to others. For example, one participant described an experience of Korean people arguing with him about the salary issue, saying,

*Some Korean people like to compare their salary and promotion. But this is not my practice in my home country…my salary is my own business. I don’t want to share. But once, some Korean people I met at the conference, they kept asking my salary…but I don’t share…they asked me to go back to my country...* (Participant #8, Counsellor, Male)

Besides salary matters [46], all participants experienced negative situations almost every month about their employment status in South Korea. For example, all participants shared that Korean people asked them to leave their region immediately as they are not South Korean or East Asian people. For example, a participant, who expressed that some random people in the community asked her to leave the region and job due to her skin colour, said,

*…almost every month, some people asked me to leave the South Korea and my job due to my skin colour…I can hear that the word dark skin and black people from their Korean language…sometimes they called me Black shit or leave the teaching job…sometimes I heard no Black people teaching…sometimes Black people should not teach out Korean kids…it was so horrible to live in this society…* (Participant #17, Teacher, Female)

At the end of 2018, South Korea had more than two million foreigners, including professional workers and international students, which was equivalent to 4% of the region’s total population [33]. Because the number of foreigners was not high, the discrimination against minorities was significant, particularly for professional workers of colour [34]. For example, many participants experienced discrimination due to their nationality and work status as education professionals in international schools and universities, in part because they were earning more than the local professionals [46]. One said,

*…all non-Korean teachers in my school and network, regardless of their skin colour and nationality, experienced discrimination due to their existence in Korea…we are all working for our living…we don’t steal. We don’t argue. But Korean people always used their fingers to point at us. They blamed us [for] stealing their jobs…* (Participant #1, Teacher, Male)

In addition to the discrimination about their teaching positions, everyone related similar discrimination issues stemming from their very existence in South Korea [35]. Many expressed that they had experienced at least five occasions of Korean people telling them to leave their city because they were not Korean. The researcher recorded a sharing, which is as follows:

*This is not a single case…I was asked by many Korean people why I am living in the city, but a hotel. Some asked me how I can rent a living unit in the building…where did I make money…am I doing something bad and illegal so I can live in an apartment? It is so rude to ask people these questions…* (Participant #3, Nurse, Female)

In short, although the social and cultural perspectives between westernised communities and South Korea are not similar, most participants felt that Korean customs and practices targeting their non-Korean identity were rude and impolite. As a result of those social issues, most participants experienced low job satisfaction and a high level of stress, both of which could have influenced their career decisions and their desire to depart from the region [33,47].

#### 3.2.2. Skin Colour, African Heritage and Nationality

Although both internal and external elements can influence the overall experiences of international school and university education professionals, most of the study’s participants expressed the opinion that the external elements of their skin colour and Black ethnicity were the reasons for the key social biases and discrimination that arose from their existence in South Korea [30,48,49,50]. The researcher categorised the biases that the participants reported, which were based on their skin colour (i.e., Black skin) and assessed how those conditions influenced their decisions, using the model of retention, turnover, and attrition. Many participants stated that even though their skin colour did not impact their work performance and personalities, South Koreans always looked down on them. One said,

*…Many children pointed at me in the park because of my looks…they laughed at me and called me a gorilla…I politely asked their parents to correct their speaking and behaviours…horribly, their parents asked me to leave the park and save the environment, because of my skin colour…* (Participant #5, Teacher, Male)

It is not uncommon for people of colour to experience discrimination and social bias because of their skin colour [51]. However, all of the study’s participants described very serious discrimination by South Koreans in response to their skin colour. First, many were asked to leave restaurants and shops because of their colour. One said,

*…Some restaurant servers and sales associates in shops asked my family to leave the stores, as they don’t want to serve Black people…I asked them am I going back to the 19th century? But as usual, Koreans pretend[ed] they d[id]n’t understand English…but they could use English to force us out…* (Participant #15, Teacher, Male)

In addition to discrimination from private companies and organisations, the participants also experienced negative issues with public services employees based on their skin colour. For example, all experienced discrimination from the immigration department when they applied for their ID card. One shared her experience, relating that the officer put on gloves before touching her documents, saying, “…East Asian, White, Hispanic applicants, the officers did not use any gloves…but in my turn, she wore gloves immediately…after my process, I saw her take [off] the gloves for another East Asian applicant” (Participant #14). This is not a single case from the participant’s shared story; all of the others in the recounted similar kinds of situations—for example, when some took their driving test, the examiner wore gloves and a mask for protection. One said,

*…the driving test examiner did not even want to touch my document and always watched his hands…before he entered the car, he wore a mask and used some bad Korean words to describe my skin colour…after the exam, he said ‘the gorilla passed the exam,’ in the Korean language…* (Participant #16, Teacher, Male)

Living in social and cultural diversity is not uncommon in the world today, due to the strong evolution towards globalisation and internationalisation in our rapidly changing society, particularly in East Asia [52,53]. International schools and universities routinely recruit education professionals with varied backgrounds for teaching and instruction, and qualified Black education professionals join the profession by bringing high teaching qualifications. However, all of the participants in this study endured blaming and unfair judgments by Koreans in reaction to their African heritage and nationality. For example, all of the participants were regularly asked by Koreans in their communities how they could have gained American, Canadian, and Australia citizenship since they were from the African continent. One participant shared that several of his students’ parents asked him about the process of immigration into the United States. The education professionals described such questioning,

*Some parents asked me how to gain the American citizenship as they want to send their kids to the US…all they asked is because they believed I am from Africa…they even asked if my American citizenship was legal or not…did I enter the US illegally and gain citizenship by something?* (Participant #11, Teacher, Male)

Another participant reported that his landlord followed a similar line of questioning about his citizenship and African heritage every time they met. His landlord always rejected his American citizenship and accused him of using a fake document to obtain citizenship. He said,

*My landlord always asked me when will I go back to Nigeria, as I look African…I explained to him that there [have been] African American people in the US for nearly 300 years…but he pretended he did not understand the US history and continued to [tell] me to go back to Nigeria and sometimes Liberia.* (Participant #6, Teacher, Male)

In short, from the social and cultural perspectives of the South Korean people, their questions about salary, career development, skin colour, nationality, and family background could be considered natural due to their ideas of collectivism. However, some of their ways of expressing themselves and some lines of questioning can exceed the acceptable bottom line of international professionals who do not understand how to deal with the local Korean customs and practices. As a result of the foreign professionals’ negative experiences and life stories, many developed a very low degree of job satisfaction and decided to leave the region after their contracts expired [29,54,55,56].

The current study, using the approach of stress, burnout, career decision, and suicide and the lens of internal and external elements of social and cultural biases and discrimination, allowed the researcher to investigate the connections among the individuals’ physical or personal attributes, job attributes, perceptions of their workplace, job satisfaction, and their career decisions as a complex network, with focuses on their personal beliefs, opinions, sense of belonging, their sense-making process, and their personal experiences of the internal and external elements [29,54,55,56]. Based on their descriptions of their experiences, most affirmed that the external elements from South Korean society had a significant negative effect of influencing their job satisfaction and career decisions.

More importantly, although the school administrators have designed and polished their human resource management and teachers’ professional development training in an effort to increase the professionals’ sense of belonging and job satisfaction, the external elements of discrimination and social biases continued to impact the professionals’ turnover rates and decisions to leave. As a result, even though the schools have contributed additional resources to reform their management, no significant forward steps can be taken because of the external and uncontrollable factors of society [29,54,55,56].

## 4. Conclusions

### 4.1. Limitations and Future Research Directions

Every study has its own limitations. The current study only interviewed Black education professionals who, at the time, were working or had left the profession. The voices and contributions cannot cover other professional staff and personnel within the industry; future researchers should, therefore, study participants from a wider range of occupations in order to seek a more comprehensive picture of this issue. 

Second, this research was conducted in South Korea, which is one of the smallest regions in the Asian-Pacific area. Therefore, the data from foreign professionals in South Korea cannot cover most of the situations and issues in the region, and future research studies should be expanded to cover other countries with larger populations.

Third, the participants in this study were Black professionals. Given the high levels of social biases and discrimination faced by such professionals in South Korea, it is not hard to assume professionals from different backgrounds may encounter different levels of stress, burnout, and suicide issues in this region. Future research should aim to seek and explore the situations of non-Black foreign professionals in South Korea.

Fourth, some scholars may argue against the limited literature review for Black people’s living experiences in South Korea. The current study is one of the very first studies about Black people’s experiences and social problems in South Korea. Therefore, it was difficult to locate related literature reviews and studies about Black people’s mental issues in the South Korean educational environment. Therefore, the researcher could only capture related literature reviews and studies with wider perspectives. Future research studies may expand Black people’s experiences in South Korea and other East Asian nations and cities.

### 4.2. Implementation

Every study has its implications for practice. First, as mentioned above, a number of international professionals have decided to come to South Korea for professional development. This study serves as a blueprint for South Korean policymakers, government leaders, department heads, and human resources planners to reform their current planning for foreigners’ well-being, rights, and social justice. Second, due to the impacts of globalisation, professionals may seek career and professional development internationally. Based on the findings of this study, there is room for the South Korean government, school leaders, parents, and the general public to understand the rich experiences of individuals from different parts of the world. Therefore, it is necessary to reform the current social justice, education, health, and social care agendas and to develop relevant promotional plans. Through this study, a light is shone on areas where there is room for improvement. Particularly, this study explores several negative experiences and feedback from Black individuals in South Korea. The related agencies and professionals can polish and reform their multicultural policies and programmes in order to upgrade the living experiences of expatriates in South Korea.

### 4.3. Conclusions

In this study, all participants expressed a number of life stories and experiences about how internal and external elements influenced and impacted their levels of stress, burnout, suicide, and career decisions as foreign education professionals at one of the international schools and universities in South Korea. 

It is worth noting that most of the participants experienced negative internal elements from their school administrators, leaders, parents, and students due to their positions, roles, skin colour, and nationalities; such social bias and discrimination came mainly from Korean nationals. Although workplace bullying and unfairness happen in all workplace environments, stress and burnout issues developed, which influenced their suicidal behaviour and career decisions. However, based on the results, we see that internal elements are not the biggest issue. External elements emanating from society, communities, and surrounding areas were the most significant sources of participants’ negative life stories. Impolite and downright rude behaviour by Korean locals always caused significant levels of stress and burnout. All participants found that South Koreans discriminated against them based on their nationalities, skin colour, and positions, which are all unchangeable elements. Therefore, the results show that although both internal and external elements influenced workplace stress, mental health, and career outcomes of these foreign professionals in South Korea, external elements were found to be the main sources of stress among these foreign professionals. 

## Figures and Tables

**Table 1:** Demography.

Name	Position	Gender	Continent (Origin)
1	Teacher	Male	North America
2	Nurse	Female	Oceania
3	Nurse	Female	North America
4	Teacher	Female	North America
5	Teacher	Male	Africa
6	Teacher	Male	North America
7	Counsellor	Male	Africa
8	Counsellor	Male	Oceania
9	Teacher	Female	North America
10	Teacher	Female	North America
11	Teacher	Male	North America
12	Nurse	Female	Oceania
13	Teacher	Female	Europe
14	Counsellor	Female	Europe
15	Teacher	Male	Africa
16	Teacher	Male	Africa
17	Teacher	Female	Africa
18	Nurse	Female	North America

**Table 2:** Themes and subthemes.

Themes and Subthemes	
3.1	Internal Stress: Unfairness toward Foreign Professionals
3.1.1	Stress and Burnout Based on Parental Behaviours
3.1.2	Stress and Burnout Based on Behaviours from Co-Workers and Administrators
3.2	External stress: Discrimination and Social Biases against Status and Identity
3.2.1	Taking Away Employment Opportunities and Disapproval Towards Foreigners
3.2.2	Skin Colour, African Heritage and Nationality

## Appendix A

Interview Protocol Questions: The First Interview

(1) How would you describe your teaching/career/job experiences in South Korea? Please tell me more.
(2) How would you describe your teaching/career/job experiences in your school in South Korea? Please tell me more.
(3) How would you describe your living experiences in South Korea? Please tell me more.
(4) Do you enjoy your teaching/career/job experiences in South Korea? Are there any remarkable experiences? May you please share with me? How were the experiences?
(5) Follow-up questions.

Interview Protocol Questions: The Second Interview

(1) As a Black individual, teacher, nurse, counsellor, how would you describe your experiences in South Korea? Please tell me more.
(2) Did you experience any remarkable experiences as a Black individual, teacher, nurse, counsellor in South Korea? If so, may you please share with me? Please tell me more.
(3) How would you describe your skin colour as a Black individual, teacher, nurse, counsellor in South Korea? Please tell me more.
(4) Follow-up questions.
